# Efficacy and safety of first-line PD-1/PD-L1 inhibitor in combination with CTLA-4 inhibitor in the treatment of patients with advanced non-small cell lung cancer: a systemic review and meta-analysis

**DOI:** 10.3389/fimmu.2025.1515027

**Published:** 2025-02-06

**Authors:** Huimin Zhao, Shanshan Huang, Jianyu Wu, Yanlan Lu, Yue Zou, Haijian Zeng, Chunlan Li, Jin Wang, Xiaochen Zhang, Siliang Duan, Weiming Liang

**Affiliations:** ^1^ The First Affiliated Hospital of Guangxi University of Science and Technology, Guangxi University of Science and Technology, Liuzhou, Guangxi, China; ^2^ Medicine College, Guangxi University of Science and Technology, Liuzhou, Guangxi, China

**Keywords:** PD-1 inhibitor, programmed cell death protein 1 inhibitor, CTLA-4 inhibitor, non-small cell lung cancer, overall survival, objective response rate, meta-analysis

## Abstract

**Introduction:**

The combination of PD-1/PD-L1 inhibitor with CTLA-4 inhibitor for advanced non-small cell lung cancer(NSCLC) is presently a significant area of research, however its clinical application remains contentious. This meta-analysis aimed to assess the efficacy and safety of first-line PD-1/PD-L1 inhibitor in combination with CTLA-4 inhibitor (CP) in the treatment of patients with advanced NSCLC.

**Methods:**

A systemic search was conducted in four databases (PubMed, Cochrane library, Embase, and Web of Science) from their establishment until January 17, 2024, for randomized controlled trials that investigated the use of the first-line PD-1/PD-L1 inhibitor plus CTLA-4 inhibitor in patients with advanced NSCLC. Progression-free survival (PFS), overall survival (OS), objective response rate (ORR), disease control rate (DCR), and adverse events (AEs) were subjected to meta-analyses.

**Results:**

Totally 7 eligible randomized controlled trials including 4682 people were included. Two comparative analyses were performed: CP versus chemotherapy, CP versus PD-1/PD-L1 inhibitor (P). Compared with the chemotherapy group, CP improved OS (HR: 0.84, 95% CI: 0.75-0.94, p<0.05) but not PFS (HR: 0.94, 95%CI: 0.73-1.20, p = 0.63) or ORR (OR: 1.16, 95% CI: 0.79-1.71, p = 0.45). In terms of toxicity, CP had slightly fewer any AEs compared to chemotherapy (RR: 0.94, 95% CI: 0.91-0.97; p<0.05). Compared to the P group, there was no significant difference in OS (MD: -0,25, 95% CI: -2.47-1.98, p = 0.83), PFS (MD: -0.91, 95% CI: -3.19-1.36, p = 0.43), and ORR (OR:1.05, 95% CI. 0.80-1.36, p = 0.73). Subgroup analysis revealed that CP provided superior OS compared with P in patients with PD-L1 expression < 1%.

**Conclusion:**

CP was a feasible and safe first-line therapy for patients with advanced NSCLC. Specifically, CP may function as a therapeutic alternative for individuals with low or negative PD-L1 expression, resulting in enhanced long-term outcomes compared to chemotherapy or P. Further randomized controlled trials with prolonged follow-up periods are necessary to validate these results, particularly focusing on efficacy in patients with differing PD-L1 expression levels, to improve the stratified implementation of immunotherapy.

**Systematic review registration:**

https://www.crd.york.ac.uk/prospero/display_record.php?ID=CRD42024621116, identifier CRD42024621116.

## 1 Introduction

A recent publication from the World Health Organization’s International Agency for Research on Cancer indicates that lung cancer is the predominant malignant tumor globally regarding incidence and fatality rates (1). According to Cancer Statistics 2024 (2), although the incidence of lung cancer has declined in the United States, with lung cancer deaths in men down 59% from the 1990 peak and in women down 36% from the 2002 peak, lung cancer still causes far more deaths each year than colorectal, breast, and prostate cancers combined. Lung cancer is histologically categorized into small cell lung cancer and non-small cell lung cancer (NSCLC), the latter representing over 85 percent of all instances. The predominant histological subtype of NSCLC worldwide is adenocarcinoma (40%), succeeded by squamous carcinoma (25%). Due to the subtlety of early lung cancer symptoms, approximately one-third of patients are diagnosed at an advanced stage, so missing the optimal window for drastic surgical intervention. Age-standardized 5-year relative/net survival rate of lung cancer was typically low, with 10%-20% for most regions (3).

The advent of immunotherapy has introduced a novel approach for the treatment of advanced NSCLC, marking the onset of the immunotherapy era in lung cancer management. Immunotherapy for NSCLC often employs various antibodies to inhibit the identification of antigens by immune cells and ligands in tumor cells. Immune checkpoint inhibitors (ICIs) have emerged as the primary treatment for advanced and metastatic NSCLC. The predominant targets in NSCLC are cytotoxic T lymphocyte-associated protein 4 (CTLA-4), programmed death receptor 1 (PD-1), and programmed death ligand 1 (PD-L1) (4). PD-1 and its ligands, PD-L1 and PD-L2, are essential regulators of immunosuppression within the local tumor microenvironment (TME). The PD-1 receptor is prominently expressed in fatigued T cells, particularly tumor-infiltrating lymphocytes. The PD-L1 receptor is abundantly expressed in all tumor cell types, while the PD-L2 receptor is only expressed on activated dendritic cells and some macrophages. PD-1/PD-L1 inhibitors can rejuvenate T lymphocytes from a dysfunctional condition by obstructing the interaction between PD-1 and PD-L1, so effectively eliminating tumor cells (5). CTLA-4 is an immunological checkpoint molecule mostly expressed on activated T cells and regulatory T (Treg) cells, which prevents T cell activation and maintains immune homeostasis. The interaction of CTLA-4 with the B7 molecule induces T cell anergy and contributes to the negative control of the immunological response. CTLA-4 inhibitor impedes T cell proliferation by obstructing the binding to B7 and disrupting the interaction between B7 and CD28. The monoclonal antibody reduces T cell proliferation by obstructing the binding to B7 and disrupting the interaction between B7 and CD28 (6).

The advancement of immunotherapy reveals that dual immunotherapy offers novel approaches for lung cancer treatment. Considering that PD-1 and CTLA-4 modulate effector T-cell activation, proliferation, and function through distinct yet complementary pathways, the application of dual immune checkpoint inhibitors (PD-1/PD-L1 and CTLA-4) is a rational approach to augment antitumor immunity (7, 8). The CheckMate227 (9) trial reported 5-year follow-up data, regardless of the patient’s PD-L1 expression level, compared with chemotherapy, nivolumab combined with ipilimumab could bring durable and long-term survival benefits. In patients with PD-L1 ≥ 1%, the 5-year OS rate of dual antibody was 24%, while it was 19% in patients with PD-L1 < 1%. It seems that the efficacy is different in patients with varying levels of PD-L1 expression. Besides, the combination of PD-1/PD-L1 and CTLA-4 for advanced non-small cell lung cancer is currently a prominent research focus, although its clinical applicability has not achieved consensus.

This meta-analysis aimed to assess the efficacy and safety of first-line PD-1/PD-L1 inhibitor in combination with CTLA-4 inhibitor in the treatment of patients with advanced NSCLC. To be more specific, this meta-analysis specified the particular advantages of dual immune therapy compared to monotherapy or chemotherapy, and conducted stratified analyses on patients with different PD-L1 expression levels to explore the potential for personalized treatment.

## 2 Materials and methods

The present meta-analysis was conducted in accordance with the Preferred Reporting Items for Systematic Review and Meta-Analysis standards (PRISMA). This meta-analysis has been formally registered at PROSPERO with the registration number CRD42024506196.

### 2.1 Search strategy

A comprehensive search was conducted in four databases: PubMed, Cochrane library, Embase, and Web of Science, covering all pertinent literature published from the library’s inception until January 17, 2024. The search was conducted using “subject + free word”, and the search terms included: “Non-Small Cell Lung Cancer”, “PD-1/L1 inhibitors” and “randomized controlled trial”. **Supplementary Table S1** provided a comprehensive listing of the search results.

### 2.2 Selection criteria

The criteria for inclusion were as follows: (1) patients diagnosed with advanced non-small cell lung cancer (NSCLC) verified by pathology or imaging, and classified as NSCLC stage III-IV according to the TNM staging of lung cancer; (2) patients in the intervention group received PD-1/PD-L1 inhibitor in combination with CTLA-4 inhibitor as their first-line treatment; (3) patients in the controlled group received other therapy as their first-line treatment; (4) at least one of the following outcomes were reported: ORR, PFS, OS, AEs, and irAEs (10). (5)Study design: randomized controlled trials (RCTs).

The criteria for exclusion were as follows: (1) other types of articles, such as case reports, publications, letters, comments, reviews, meta-analyses, editorials, animal studies, protocols, conference, etc.; (2) other types of diseases; (3) patients with EGFR or ALK genetic mutation, since tyrosine kinase inhibitors have become the first-line standard choice for these patients (11–16); (4) phase I trials; (5) single arm experiment; (6) data cannot be extracted; (7) duplicate patient cohort.

### 2.3 Data extraction

Two reviewers independently extracted the following pertinent data: first author, publication year, country, participant count, participant characteristics (median age, gender, stage of NSCLC, and performance status), treatment arm. The measures of antitumor effectiveness (OS, PFS and ORR) and toxicity (adverse events) were obtained. Any disagreements were settled by another reviewer. A subgroup analysis based on PD-L1 expression was performed. Patients were categorized into three groups: negative (<1%), low-positive (1-50%), and high-positive (>50%). Besides, analyses were performed based on blood tumor mutational burden (TMB), medication combinations, non-small cell lung cancer stage, median age, gender, histology and smoking status.

### 2.4 Quality assessment

The quality of the 7 included trials was evaluated using the risk of bias assessment tool implemented by the Cochrane Collaboration (17). The evaluation included the following seven crucial areas: (1) technique of randomization; (2) concealment of the allocation scheme; (3) blinding of participants and administrators of the treatment protocol; (4) blinding of outcome measurements; (5) completeness of outcome data; (6) selective reporting of research outcomes; and (7) additional sources of bias. All studies were assessed to be at risk in each of these seven domains. Upon examination according to the aforementioned criteria, each study was classified into high-quality, medium-quality, and low-quality categories based on the evaluation results.

### 2.5 Statistical analysis

The meta-analysis was conducted using Review Manager (Revman5.3) and Stata (version 17.0) for data analysis. The effect size for OS and PFS was identified as HR (18) or MD, the effect size for ORR was identified as OR, while the effect size for incidence of adverse events, and AEs was identified as RR. The effect sizes were visualized using forest plots. The heterogeneity test was evaluated using the Q test (19) (Q test, Chi square test), and quantitatively determined using I^2^. For I² values less than 50%, a fixed effect model was employed, whereas for I² values more than 50%, a random effect model was included in the study. When there is heterogeneity in the results, in order to analyze its source, subgroup analysis can be carried out, divided into two subgroups according to the type of study, when the two groups are homogeneous within the group, and there is heterogeneity in the results after combining them, it indicates that the type of study may be the source of the heterogeneity; or sensitivity analysis is carried out, adopting article-by-article elimination method, which is carried out by deleting the studies one by one to observe whether the heterogeneity is changed or not, and if the results are consistent before and after, it indicates that the results of performing if the results are consistent, it indicates that the results of the random effects calculation are stable and reliable. Discrepancies in the results of the sensitivity analysis suggest that the findings lack robustness and should be interpreted with caution. To evaluate the existence of publication bias, a funnel plot was constructed. The presence of symmetrical two sides in the funnel plot suggests the absence of evident publishing bias. Conversely, asymmetrical two sides of the funnel plot hint the potential existence of publication bias. Statistical significance was declared when the p-value was less than 0.05.

## 3 Results

### 3.1 Search results and study quality assessment

**Figure 1:**
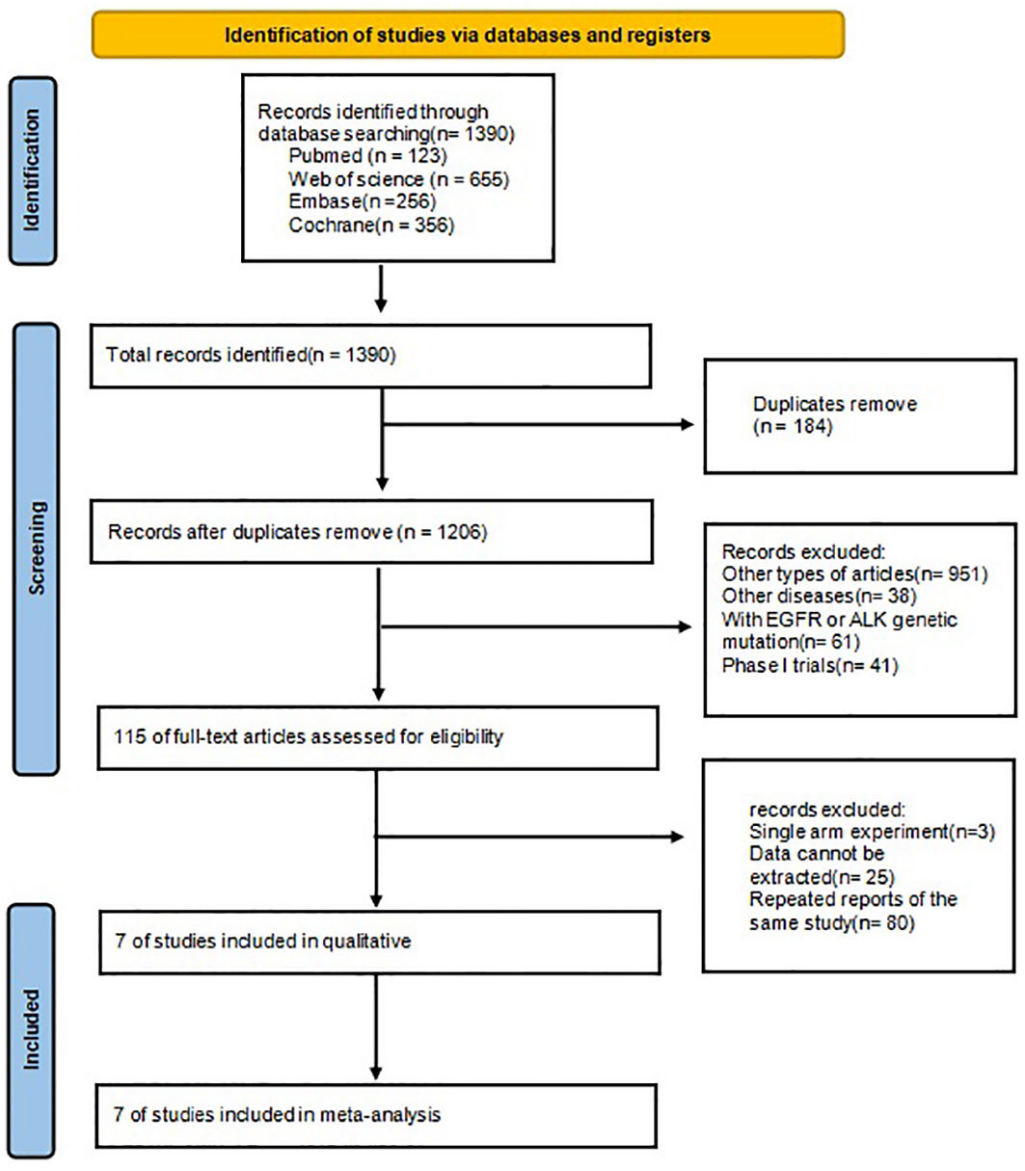
Flow chart of literature search strategies.

The procedure for selecting and integrating the literature is shown in **Figure 1**. A total of 1390 records were initially identified. After eliminating duplicate studies, a total of 1206 papers remained. Based on the assessment of titles and abstracts, a total of 115 papers were eliminated. After a thorough review of the full text, 7 studies were selected for inclusion in this meta-analysis.

### 3.2 Patient characteristics

This meta-analysis comprised 7 RCTs (20–26) published between 2020 and 2023. These trials included a total of 4682 patients who were diagnosed with advanced non-small cell cancer. The authors, year, trial name, disease stage, study period, group, treatment arms, cases, median age, male, OS and PFS of the included literature are shown in **Table 1**.

**Table 1:** Characteristics of included studies.

Author (year)	Trial name	Disease stage	Study period	Group	Treatment arms	Cases	Median age	Male (%)	OS (95% CI)	PFS (95% CI)
D. Planchard 2020 (25)	ARCTRC	IIIB/IV	2015-2016	PD-L1 TC ≥25%	Arm 1: D 10 mg/kg q2w	62	63.5	67.7	11.7 (8.2—17.4)	3.8 (1.9—5.6)
					Arm 2: SOC	64	62	75	6.8 (4.9—10.2)	2.2 (1.9—3.7)
				PD-L1 TC ≤ 25%	Arm 1: D 20 mg/kg q4w + T 1 mg/kg q4w	174	62.5	66.1	11.5 (8.7—14.1)	3.5 (2.3—4.6)
					Arm 2: SOC	118	65	68.6	8.7 (6.5—11.7)	3.5 (1.9—3.9)
					Arm 3: D 10 mg/kg q2w	117	63	62.4	10.0 (7.1—13.2)	3.1 (1.9—3.7)
					Arm 4: T 10 mg/kg q4w	60	63.5	65	6.9 (3.9—13.2)	2.1 (1.8—3.2)
Michael Boyer 2021 (20)	KEYNOTE-598	IV	2018-2019	Arm 1	P 200mg plus I 1mg/kg q6w	284	64	71.1	21.4 (16.6 to NA)	8.2 (6.0 to 10.5)
				Arm 2	P 200mg plus placebo	284	63	67.3	21.9 (18.0 to NA)	8.4 (6.3 to 10.5)
Luis Paz-Ares 2021 (24)	CheckMate9LA	IV	2017-2019	Arm 1	N 360 mg q3w + I 1 mg/kg q6w + chemotherapy (every 3 weeks for two cycles)	361	65	70	14·1 (13·2–16·2)	6·8 (5·6–7·7)
				Arm 2	chemotherapy	358	65	70	10·7 (9·5–12·4)	5.0 (4·3–5·6)
Natasha B. Leighl 2022 (23)	CCTG BR34	IV	2017-2018	Arm 1	D 1500 mg q3w + T 75 mg q3w + chemotherapy q3w	151	65	53.6	16.6 (12.6—19.1)	7.7 (5.5—8.5)
				Arm 2	D 1500 mg q4w + T 75 mg q4w	150	63	54	14.4 (10.6—18.3)	3.2 (2.7—5.1)
Ying Cheng 2023 (21)	NEPTUNE	IV	2017-2018	Arm 1	D 20 mg/kg q4w + T1 mg/kg q4w	78	61	76.9	20.0 (15.0—28.7)	4.2 (2.8—7.2)
				Arm 2	chemotherapy q3w	82	62.5	69.5	14.1 (9.5—19.4)	6.0 (5.5—7.5)
Naiyer A. Rizvi 2020 (26)	MYSTIC	IV	2015-2016	Arm 1	D 20 mg/kg q4w	374	63.2	68.4	16.3 (12.2-20.8)	4.7 (3.1-6.3)
				Arm 2	D 20 mg/kg q4w + T 1 mg/kg q4w	372	64.3	71.5	11.9 (9.0-17.7)	3.9 (2.8-5.0)
				Arm 3	chemotherapy	372	63.6	67.2	12.9 (10.5-15.0)	5.4 (4.6—5.8)
M.D. Hellmann 2019 (22)	CheckMate227	IV	2015-2016	Arm 1	N 3 mg/kg q2w + I 1 mg/kg q6w	583	64	67.4	17.1 (15.2–19.9)	5.2 (4.1–6.6)
				Arm 2	chemotherapy q3w	583	64	66	13.9 (12.2–15.1)	5.6 (5.3–6.7)

D, durvalumab; T,tremelimumab; D + T, durvalumab + tremelimumab; P, pembrolizumab; P + I, pembrolizumab + ipilimumab; N + I, nivolumab + ipilimumab; SoC regimens, erlotinib, gemcitabine,or vinorelbine. NA, not available.

### 3.3 Risk of bias

**Figure 2:**
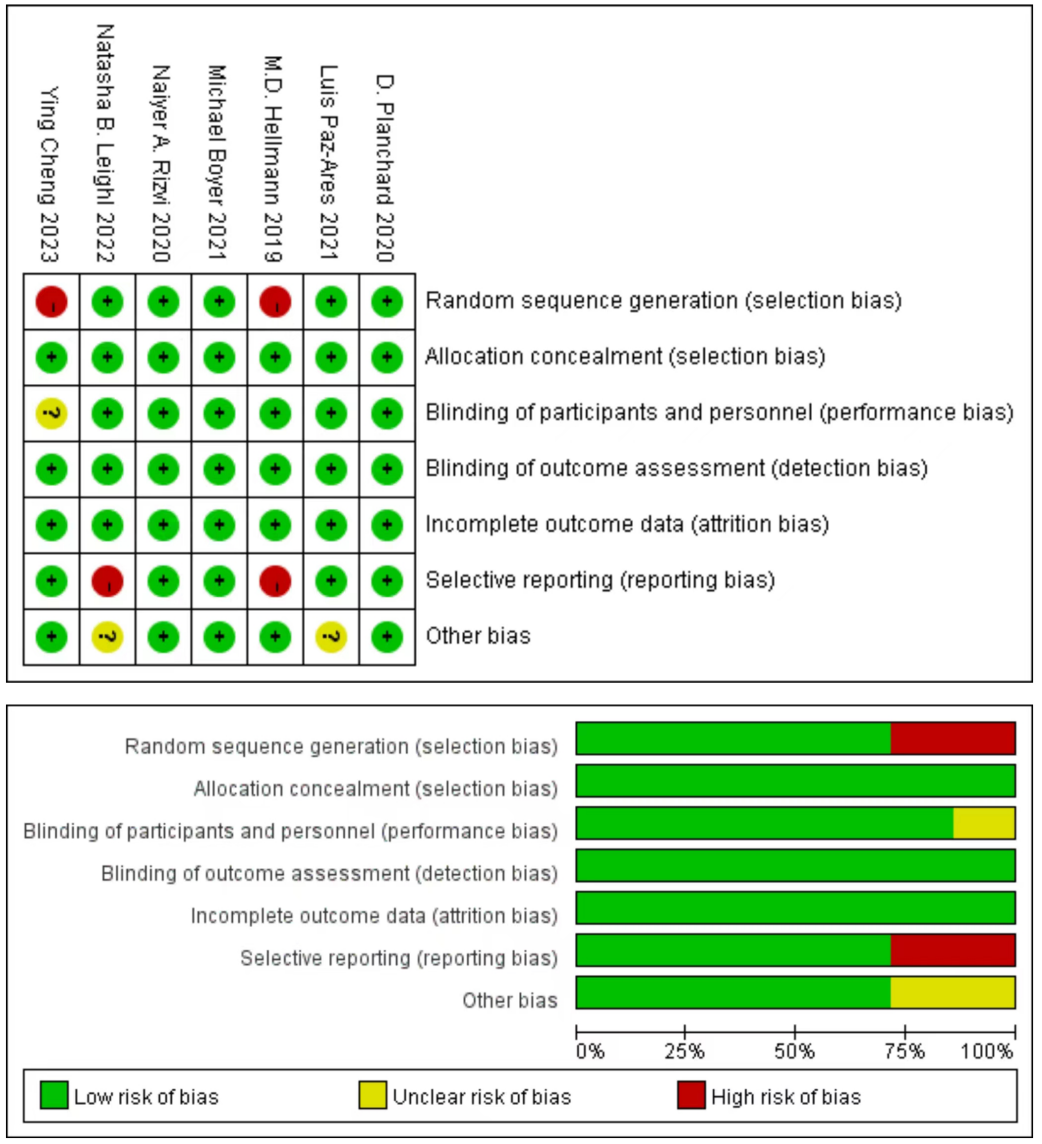
Risk of bias assessment diagram.

Cochrane Risk of Bias Tool evaluation indicated that the included literature consisted of studies of medium to high quality, five studies produced a sufficient random sequence, seven studies reported appropriate allocation concealment, six studies clearly implemented participant blinding, seven studies reported outcome assessor blinding, seven studies provided complete outcome data, five studies did not engage in selective reporting, and five studies did not exhibit any other bias (**Figure 2**).

### 3.4 CP versus chemotherapy

**Figure 3:**
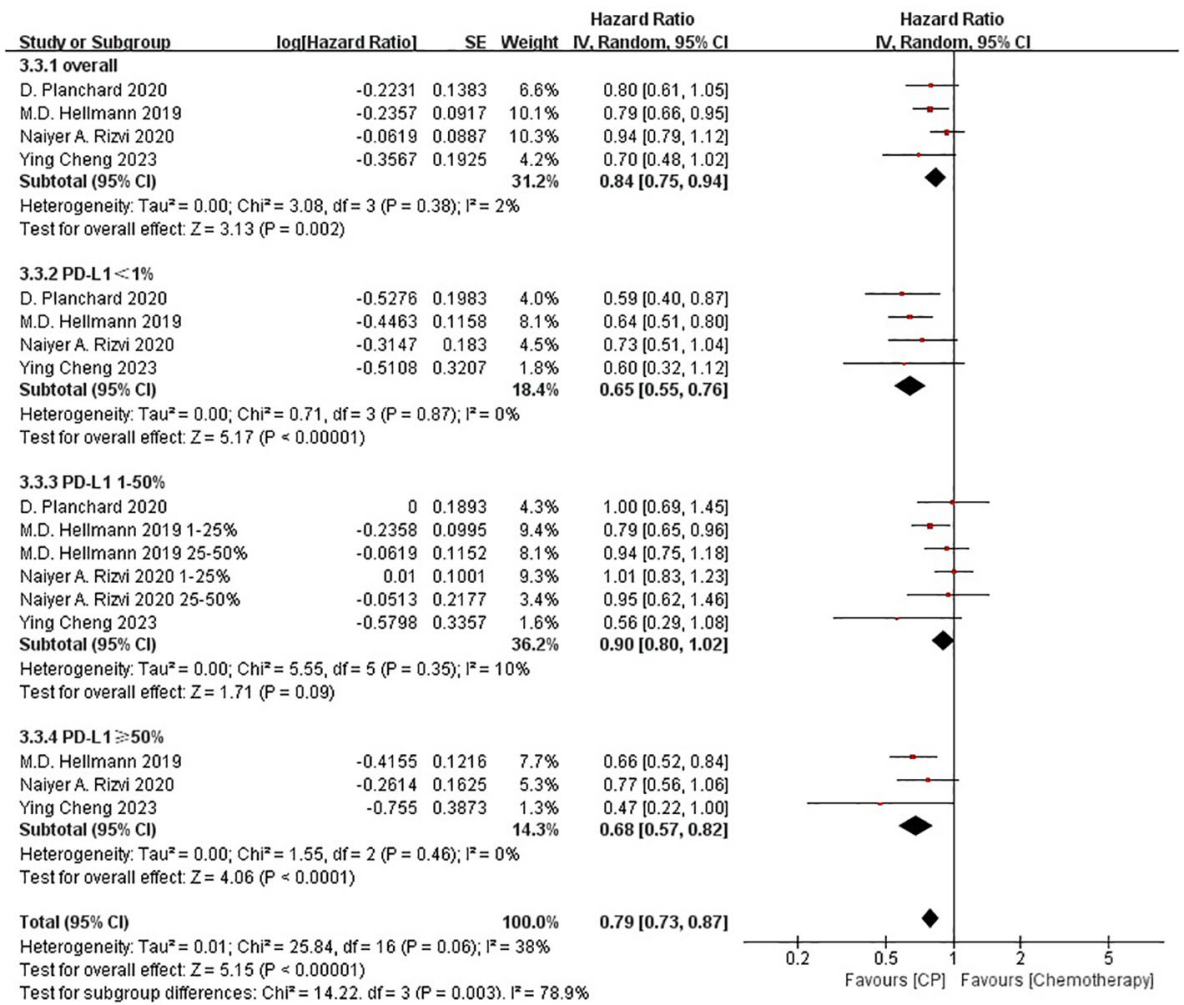
Forest plot of the meta-analysis for OS (CP vs chemotherapy).

Four studies (21, 22, 25, 26) compared the OS between the CP group and the chemotherapy group (heterogeneity: p = 0.38, I^2^ = 2%). The results indicated that the CP provided significantly better OS compared with chemotherapy(HR: 0.84, 95% CI: 0.75-0.94, p <0.05) (**Figure 3**).

**Figure 4:**
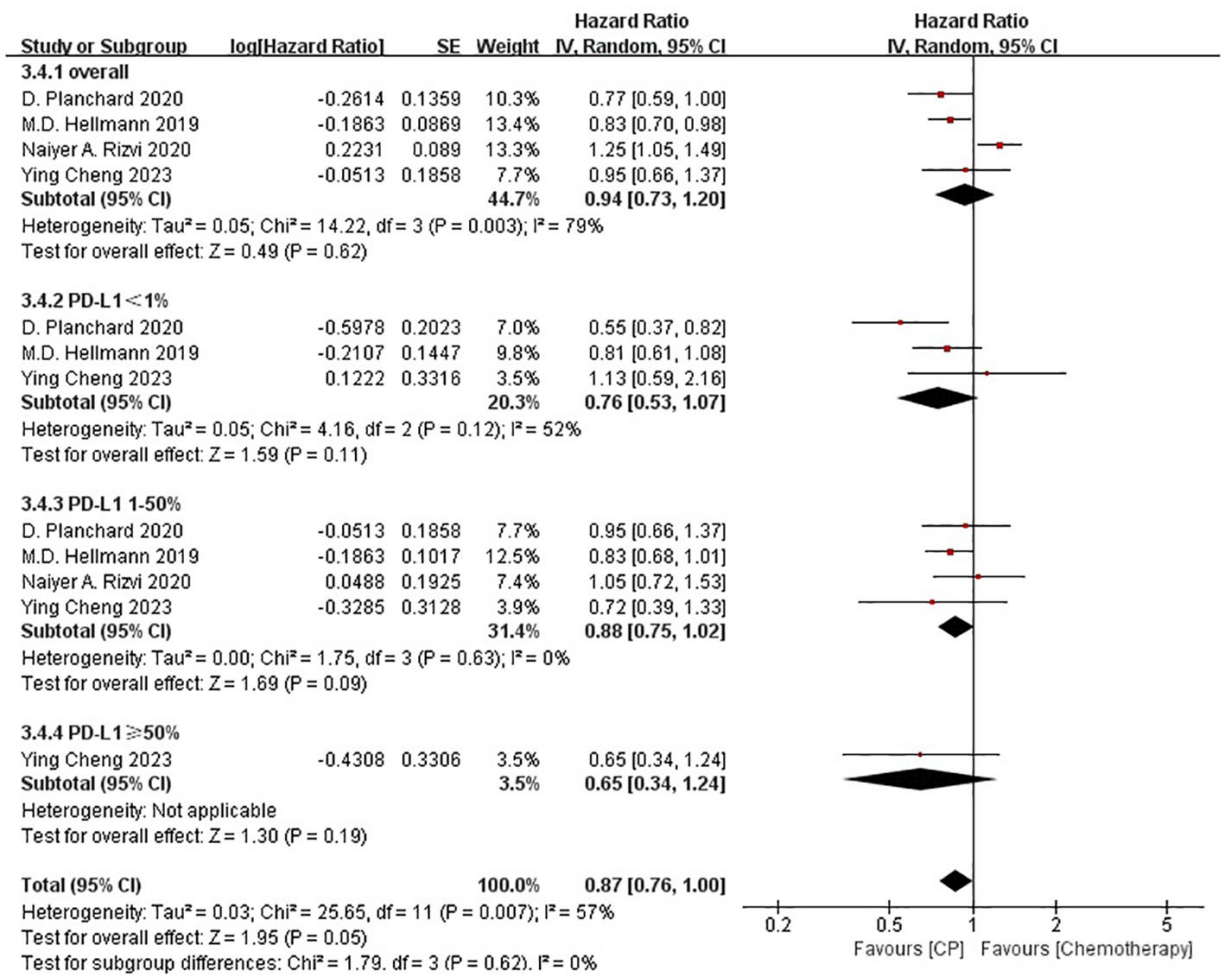
Forest plot of the meta-analysis for PFS (CP vs chemotherapy).

Four studies (21, 22, 25, 26) compared the PFS between the CP group and the chemotherapy group (heterogeneity: p = 0.003, I^2^ = 79%). The results indicated no significant difference in PFS between the CP group and the chemotherapy group(HR: 0.94, 95% CI: 0.73-1.20, p = 0.62) (**Figure 4**).

**Figure 5:**
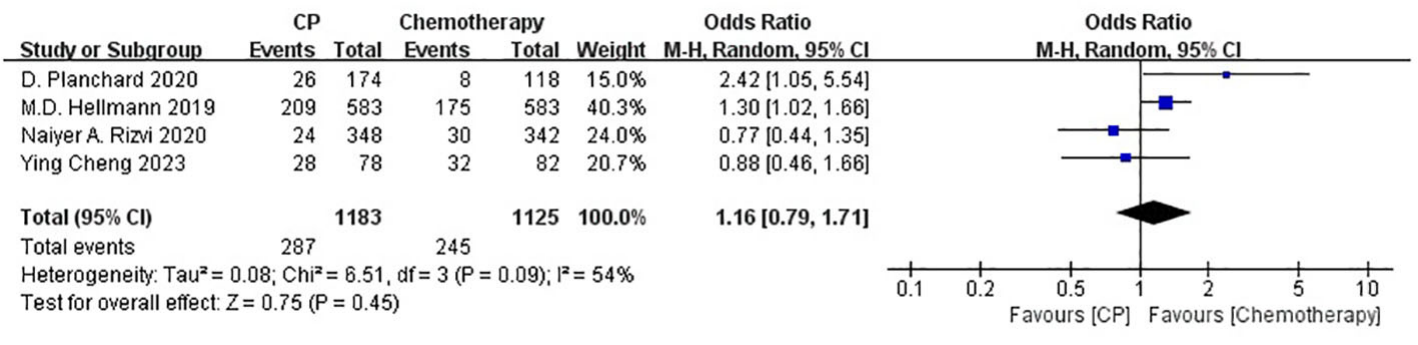
Forest plot of the meta-analysis for ORR (CP vs chemotherapy).

Four studies (21, 22, 25, 26) compared the ORR between the CP group and the chemotherapy group(heterogeneity: p *=* 0.09, I^2^ = 54%). The results indicated no significant difference in ORR between the CP group and the chemotherapy group(OR: 1.16, 95% CI: 0.79-1.71, p = 0.45) (**Figure 5**).

The non-significant results regarding PFS and ORR may be affected by sample size or heterogeneity. Therefore, subgroup analyses were conducted regarding PD-L1 expression, blood TMB, medication combinations, tumor stage, median age, gender, histology and smoking status (**Table 2**). It was worth noting that, CP provided significantly better OS compared with chemotherapy while PD-L1 expression was less than 1% or greater than 50%, or bTMB over 20 mut/Mb.

**Table 2:** Results of the meta-analysis for OS and PFS (CP vs chemotherapy).

Group	Overall survival				Progression-free survival				
	No.of studies	HR (95%CI)	P	I^2^ (%)	No.of studies	HR (95%CI)	P		I^2^ (%)
CTLA4 inhibitor+PD1/PD-L1 inhibitor VS chemotherapy									
Total	4	0.84 (0.75,0.94)	<0.05	2	4	0.94 (0.73,1.20)	0.62		79
PD-L1 expression									
<1%	4	0.65 (0.55,0.76)	<0.05	0	3	0.76 (0.53,1.07)	0.11		52
1-50%	4	0.90 (0.80, 1.02)	0.09	10	4	0.88 (0.75, 1.02)	0.09		0
≥50%	3	0.68 (0.57,0.82)	<0.05	0	1	0.65 (0.34,1.24)	0.19		—
Blood Tumor Mutational Burden									
bTMB ≥20 mut/Mb	3	0.64 (0.52, 0.78)	<0.05	0	3	0.78 (0.60, 1.02)	0.07		54
bTMB<20 mut/Mb	2	0.94 (0.61, 1.43)	0.76	85	2	0.97 (0.70, 1.35)	0.87		82
Drug combination									
Nivolumab+ipilimumab	1	0.70 (0.48,1.02)	0.06	—	1	0.83 (0.70,0.98)	0.03		—
Durvalumab+tremelimumab	3	0.85 (0.76,0.96)	0.01	5	3	0.98 (0.71,1.36)	0.92		79
NSCLC stage									
IIIB/IV	1	0.80 (0.61,1.05)	0.11	—	1	0.77 (0.59,1.00)	0.05		—
IV	3	0.84 (0.72,0.98)	0.02	32	3	1.00 (0.74,1.34)	1.00		82
Median age of CP group									
≥65 years old	1	0.94 (0.79,1.12)	0.49	—	1	1.25 (1.05,1.49)	0.01		—
<65 years old	3	0.78 (0.68,0.90)	<0.05	0	3	0.83 (0.73,0.95)	0.01		0
Male, n (%) of CP group									
Male>70%	2	0.85 (0.65,1,12)	0.25	48	2	1.14 (0.89,1.47)	0.30	44	
Male<70%	2	0.79 (0.68,0.92)	<0.05	0	2	0.81 (0.70,0.94)	<0.05	0	
Histology, n (%) of CP group									
Non-squamous ≥70%	2	0.79 (0.68,0.92)	<0.05	0	2	0.81 (0.70,0.94)	<0.05	0	
Non-squamous <70%	2	0.85 (0.65,1.12)	0.25	48	2	1.14 (0.89,1.47)	0.30	44	
Smoking status, n (%) of CP group									
Current or former smoker ≥80%	3	0.85 (0.76,0.96)	0.01	5	3	0.94 (0.69,1.27)	0.67	96	
Current or former smoker <80%	1	0.70 (0.48,1.02)	0.06	—	1	0.95 (0.66,1.37)	0.78	—	

CI, confidence interval; CP, CTLA4 inhibitor + PD1/PD-L1 inhibitor; CTLA4, cytotoxic T-lymphocyte-associated protein 4; HR, Hazard ratio; NSCLC, non-small cell lung cancer; PD-L1, programmed cell death receptor ligand 1.

### 3.5 CP versus P

**Figure 6:**
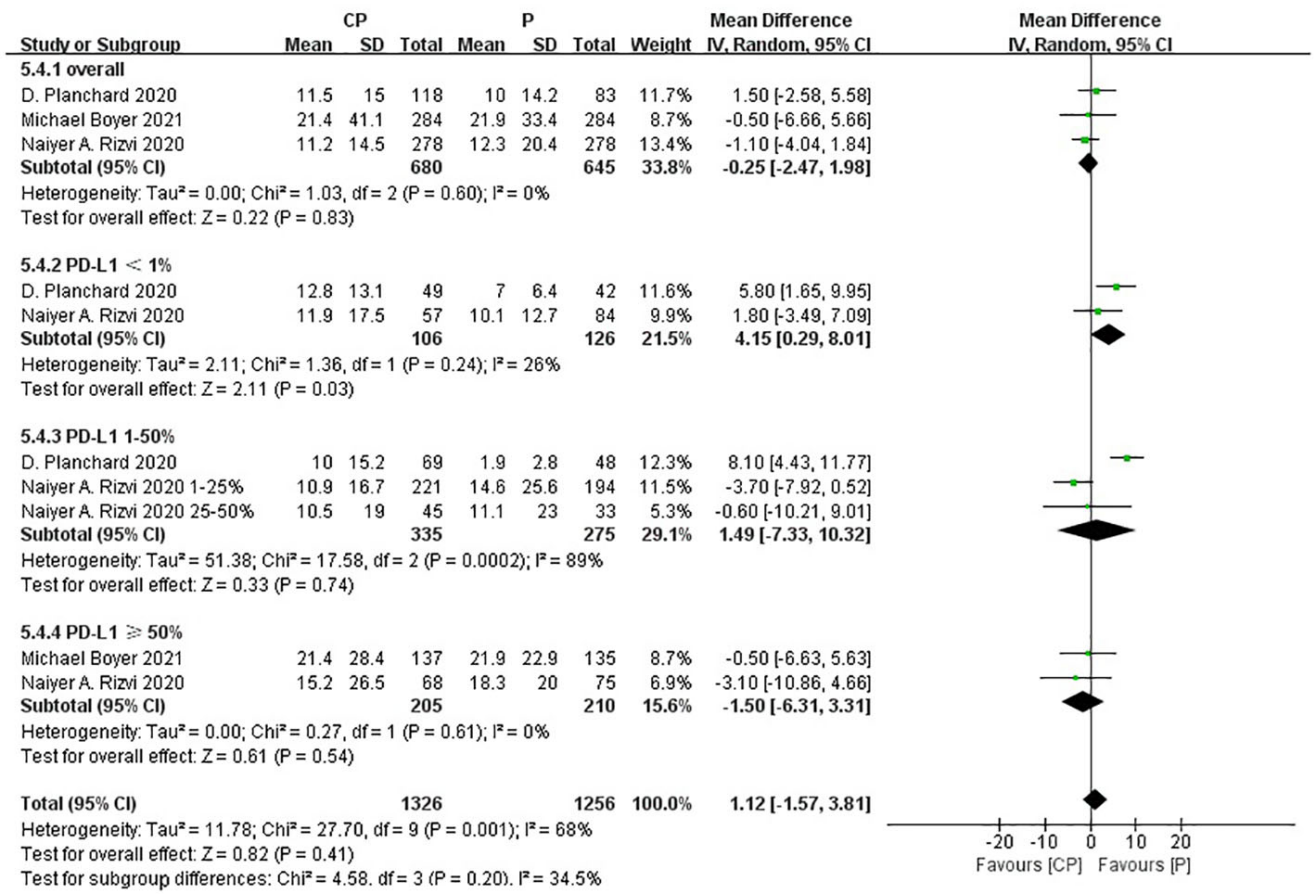
Forest plot of the meta-analysis for OS (CP vs P).

Three studies (20, 25, 26) compared the OS between the CP group and the P group (heterogeneity: p = 0.60, I^2^ = 0%). The results indicated no significant difference in OS between the CP group and the P group (MD: -0.25, 95%CI: -2.47-1.98, p = 0.83) (**Figure 6**).

**Figure 7:**
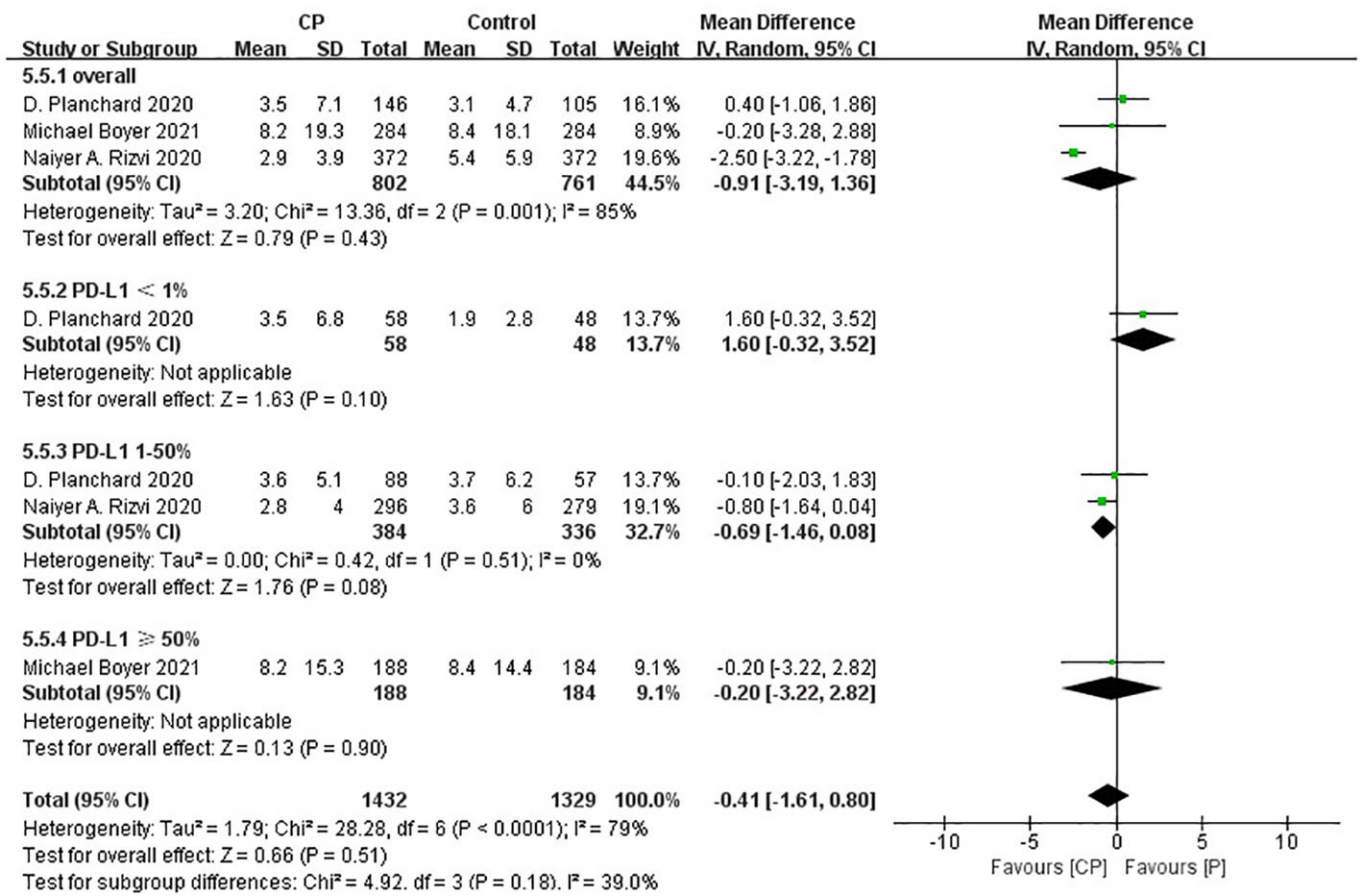
Forest plot of the meta-analysis for PFS (CP vs P).

Three studies (20, 25, 26) compared the PFS between the CP group and the P group (heterogeneity: p = 0.001, I^2^ = 85%). The results indicated no significant difference in PFS between the CP group and the P group (MD: -0.91, 95%CI: -3.19–1.36, p = 0.43) (**Figure 7**).

**Figure 8:**
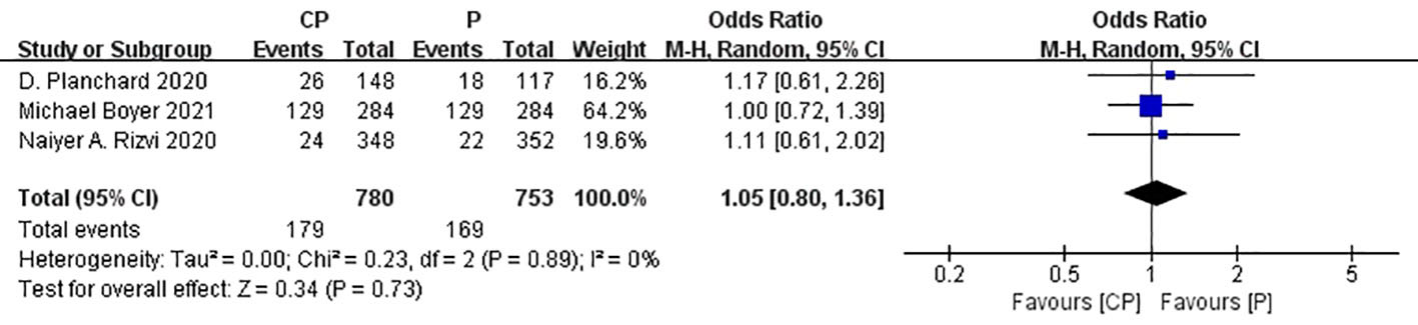
Forest plot of the meta-analysis for ORR (CP vs P).

Three studies (20, 25, 26) that compared the ORR between the CP group and the P group(heterogeneity: p = 0.89, I^2^ = 0%). The results indicated no significant difference in ORR between the CP group and the P group (OR: 1.05, 95%CI: 0.80–1.36, p = 0.73) (**Figure 8**).

The non-significant results regarding OS, PFS and ORR may be affected by sample size or heterogeneity. Therefore, subgroup analyses were conducted regarding PD-L1 expression, blood TMB, medication combinations, tumor stage, median age, gender, histology and smoking status (**Table 3**). It was worth noting that, CP provided significantly better OS compared with P while PD-L1 expression was less than 1%.

**Table 3:** Results of the meta-analysis for OS and PFS (CP vs P).

Group	Overall survival				Progression-free survival			
	No.of studies	MD (95%CI)	P	I^2^ (%)	No.of studies	MD (95%CI)	P	I^2^ (%)
CTLA4 inhibitor+PD1/PD-L1 inhibitor VS PD1/PD-L1 inhibitor								
Total	3	-0.25 (-2.47,1.98)	0.83	0	3	-0.91 (-3.19,1.36)	0.43	85
PD-L1 expression								
<1%	2	4.15 (0.29,8.01)	0.03	26	1	1.60 (-0.32,3.52)	0.10	—
1-50%	2	1.49 (-7.33, 10.32)	0.74	89	2	-0.69 (-1.46, 0.08)	0.74	89
≥50%	2	-1.50 (6.31,3.31)	0.54	0	1	-0.20 (-3.22,2.82)	0.90	—
Blood Tumor Mutational Burden								
bTMB ≥20 mut/Mb	1	9.30 (-1.89, 20.49)	0.10	—	1	1.50 (-0.11, 3.11)	0.07	—
bTMB<20 mut/Mb	1	-2.50 (-5.24, 0.24)	0.07	—	1	-0.80 (-1.47, -0.13)	0.02	—
Drug combination								
Pembrolizumab + ipilimumab	1	-0.50 (-6.66,5.66)	0.87	—	1	-0.20 (-3.28,2.88)	0.90	—
Durvalumab+tremelimumab	2	-0.20 (-2.62,2.22)	0.87	3	2	-1.12 (-3.96,1.72)	0.44	92
NSCLC stage								
IIIB/IV	1	1.50 (-2.58,5.58)	0.47	—	1	0.40 (-1.06,1.86)	0.59	—
IV	2	-0.99 (-3.64,1.67)	0.47	0	2	-1.86 (-3.88,0.17)	0.07	51
Median age of CP group								
≥65 years old	1	-1.10 (-4.04,1.84)	0.46	—	1	-2.50 (-3.22,-1.78)	<0.05	—
<65 years old	2	0.89 (-2.51,4.29)	0.61	0	2	0.29 (-1.03,1.61)	0.67	0
Male, n (%) of CP group								
Male>70%	2	-0.99 (-3.64,1.67)	0.47	0	2	-1.86 (-3.88,0.17)	0.07	51
Male<70%	1	1.50 (-2.58,5.58)	0.47	—	1	0.40 (-1.06,1.86)	0.59	—
Histology, n (%) of CP group								
Non-squamous ≥70%	2	0.89 (-2.51,4.29)	0.61	0	2	0.29 (-1.03,1.61)	0.670	0
Non-squamous<70%	1	-1.10 (-4.04,1.84)	0.46	—	1	-2.50 (-3.22,-1.78)	<0.05	—
Smoking status, n (%) of CP group								
Current or former smoker ≥85%	1	-0.50 (-6.66,5.66)	0.87	—	1	-0.20 (-3.28,2.88)	0.90	—
Current or former smoker<85%	2	-0.20 (-2.62,2.22)	0.87	3	2	-1.12 (-3.96,1.72)	0.44	92

CI, confidence interval; CP, CTLA4 inhibitor + PD1/PD-L1 inhibitor; CTLA4, cytotoxic T-lymphocyte-associated protein 4; MD, Mean Difference; NSCLC, non-small cell lung cancer; PD-L1, programmed cell death receptor ligand 1.

### 3.6 CP plus chemotherapy

Within the literature reviewed, two trials assessed the regimen of CP combined with chemotherapy: nivolumab + ipilimumab + chemotherapy (27) and durvalumab + tremelimumab + chemotherapy (28). However, we failed to performed a meta-analysis due to the different control groups in the two trials. Therefore, only a concise overview of the two clinical trials involving different control groups is presented here. Nivolumab + ipilimumab + chemotherapy compared with chemotherapy in CheckMate9LA (24) found that double-exempt combination chemotherapy with ICIs improved OS compared with chemotherapy alone (HR: 0.66, 95% Cl: 0.55-0.80), PFS(HR: 0.68, 95% CI: 0.57-0.82), but the incidence of AEs(91% versus 87%) and 3-5 AEs(47% versus 38%) was also significantly higher and toxicity was increased; in CCTG BR34 (23) durvalumab + tremelimumab + chemotherapy was compared with durvalumab + tremelimumab, and it was found that ICIs double-exempt combination chemotherapy improved PFS (HR: 0.67, 95% CI: 0.52-0.88), but OS was not prolonged (HR: 0.88, 90% CI: 0.67-1.16), and chemotherapy plus within the immunotherapy group, 82% of patients experienced grade 3 or higher adverse events, while in the control group, 70% experienced such events. These adverse events were associated with a heightened toxic response to the combination of ICIs double-elimination and chemotherapy.

### 3.7 Toxicity

The occurrence of all grade AEs in all included studies were summarized (**Table 4**, **Supplementary Table S2**); the most common AEs in the CP group were diarrhea (17.38%), decreased appetite (16.79%), fatigue (15.37%), rash (15.12%), pruritus (14.12%), nausea (12.87%), weight decreased (10.94%), asthenia (10.78%), back pain (10.24%), aspartate aminotransferase increased (9.60%), CP had lower incidence of all grade AEs (RR: 0.94, 95% CI: 0.91-0.97, p<0.05) compared to chemotherapy (heterogeneity: p = 0.12, I^2^ = 48%). In comparison to P (heterogeneity: p = 0.11, I^2^ = 54%), CP had a higher occurrence of all grade AEs (RR: 1.05, 95% CI: 1.00-1.10, p = 0.05).

**Table 4:** Results of the meta-analysis for AEs.

	CP group Vs. Chemotherapy group					CP group Vs. P group				
	CP group	Chemotherapy	RR	I^2^ (%)	*p*	CP group	P group	RR	I^2^ (%)	*p*
All grade AEs										
Overall	1004/1197	985/1110	0.94 (0.91, 0.97)	48	<0.05	655/826	572/767	1.05 (1.00,1.10)	54	0.05
Diarrhea	208/1197	119/1110	1.62 (1.32, 2.00)	10	<0.05	141/826	86/767	1.55 (1.21,1.98)	0	<0.05
Pruritus	175/1197	30/1110	5.41 (3.71, 7.89)	75	<0.05	136/826	95/767	1.36 (1.07,1.73)	0.01	0
Rash	181/1197	90/1110	1.87 (1.47, 2.36)	87	<0.05	107/826	74/767	1.39 (1.05,1.83)	0	0.02
Fatigue	184/1197	214/1110	0.81 (0.68, 0.97)	64	0.02	109/826	84/767	1.24 (0.95,1.61)	47	0.12
Decreased appetite	201/1197	234/1110	0.80 (0.68, 0.95)	62	0.01	112/826	70/767	1.48 (1.12,1.96)	0	<0.05
Nausea	154/1197	402/1110	0.37 (0.31, 0.43)	78	<0.05	85/826	60/767	1.35 (0.99,1.83)	60	0.06
Asthenia	129/1197	123/1110	0.98 (0.78, 1.24)	0	0.88	83/826	72/767	1.09 (0.81,1.46)	0	0.58
Anemia	92/1197	412/1110	0.21 (0.17, 0.26)	89	<0.05	53/826	52/767	0.97 (0.68,1.40)	0	0.88
Vomiting	69/1197	166/1110	0.40 (0.31, 0.52)	49	<0.05	44/826	37/767	1.14 (0.76,1.73)	51	0.52
Constipation	111/1197	189/1110	0.55 (0.44, 0.68)	79	<0.05	43/653	56/650	0.77 (0.53,1.10)	0	0.15
Grade 3–5 AEs										
Overall	481/1197	469/1110	0.94 (0.86, 1.04)	73	0.23	335/826	239/767	1.29 (1.14,1.47)	0	<0.05
Fatigue	24/1120	20/1032	1.09 (0.61, 1.94)	0	0.78	17/826	12/767	1.32 (0.64,2.74)	0	0.46
Pneumonia	32/1024	11/1000	2.79 (1.43, 5.45)	24	0.003	59/826	47/767	1.21 (0.84,1.76)	0	0.31
Diarrhea	26/1197	12/1110	1.95 (0.99, 3.82)	0	0.05	24/826	3/767	6.50 (2.12,19.92)	0	<0.05
Anemia	15/1197	124/1110	0.12 (0.07, 0.20)	0	<0.05	9/455	7/767	1.21 (0.47,3.13)	0.00	0.70
Neutropenia	1/1197	107/1110	0.02 (0.01, 0.07)	0	<0.05	2/455	0/398	2.47 (0.26,23.40)	0	0.43
Vomiting	4/1197	23/1110	0.18 (0.07, 0.50)	0	<0.05	4/282	1/281	3.99 (0.35,35.44)	-	-
Nausea	8/1024	20/1000	0.39 (0.17, 0.88)	0	0.02	3/653	0/650	3.98 (0.45,35.53)	0	0.22

A summary of all grade 3–5 AEs was compiled (**Table 2**, **Supplementary Table S3**); the most prevalent grade 3–5 AEs in the CP group were pneumonia (3.13%), febrile neutropenia (3.07%), diarrhea(2.17%), fatigue (2.14%), rash(1.74%), autoimmune hepatitis (1.73%), colitis (1.65%), pancreatitis (1,56%), dehydration(1.37%), anemia (1.25%). CP had comparable incidence of grade 3–5 AEs (RR: 0.94, 95% CI: 0.86-1.04, p = 0.23) compared with chemotherapy, but higher incidence of grade 3–5 AE in comparison to P (RR:1.29, 95% CI: 1.14-1.47, p<0.05) (**Table 4**, **Supplementary Table S3**).

### 3.8 Publication bias

**Figure 9:**
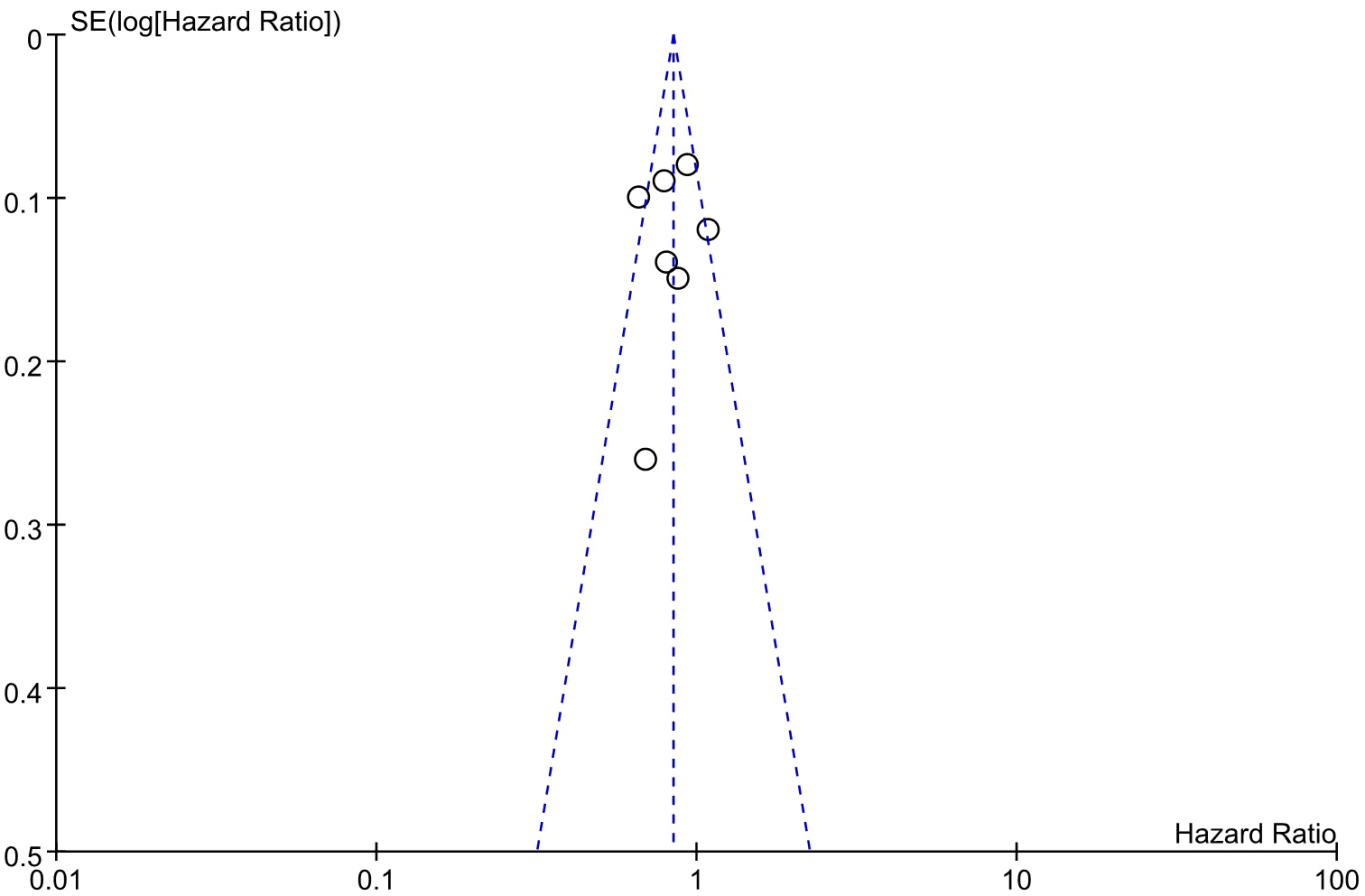
Funnel plot for OS.

An evaluation of publication bias in relation to the OS was conducted using a funnel plot (**Figure 9**). The bilateral symmetric funnel plot of the OS did not reveal any significant evidence of publication bias.

## 4 Discussion

Non-small cell lung cancer constitutes 80-85% of all lung cancers and is among the most prevalent malignant neoplasms detected globally (29). Approximately 70% of patients are diagnosed in intermediate to advanced stages of the disease (30), thereby resulting in a dismal 5-year survival rate. The contemporary approach to treating NSCLC mostly involves the use of immunosuppressants and chemotherapy. However, there is ongoing debate about the comparative effectiveness of combining two major immune checkpoint inhibitors (CTLA-4 inhibitors and PD-1/PD-L1 inhibitors) compared to using either an immunosuppressant or chemotherapy alone. This meta-analysis assessed the efficacy and safety of first-line CTLA-4 inhibitor in combination with PD-1/PD-L1 inhibitor in the treatment of patients with advanced NSCLC. Our results revealed that CP demonstrated superior efficacy, longer OS, and comparable and controllable toxicity in comparison to chemotherapy. However, there were no significant differences in total OS, PFS, and ORR compared to P, with a slightly higher toxicity.

By comparing dual immune therapy to monotherapy or chemotherapy, this meta-analysis highlighted its unique benefits. To further investigate the possibility of personalized treatment, subgroup analyses were performed on patients with varying PD-L1 expression levels. For patients with PD-L1 expression <1%, CP provided significantly longer OS in comparison to chemotherapy or P, as well as longer PFS (though no statistical difference); For patients with PD-L1 expression 1-50%, CP provided similar OS and PFS compared with chemotherapy or P; For patients with PD-L1 expression ≥50%, CP provided similar OS and PFS compared with P, but significantly longer OS in comparison to chemotherapy. In numerous trials, anti-PD-1/PD-L1 medicines have shown clinical efficacy in patients with diverse PD-L1 expression levels on tumor cells (31–35). Enhanced efficacy outcomes with PD-1/PD-L1 inhibitors have been observed in patients exhibiting higher PD-L1expression levels (31, 32, 34, 36, 37). However, low PD-L1 expression was associated with poor efficacy outcomes (36, 38, 39). Low PD-L1 expression is associated with low T-cell infiltration, resulting in a primary resistance to PD-1 inhibitors (40–42). Despite promising results from extensive clinical trials, no definitive optimal treatment existed for people with PD-L1-negative NSCLC. The findings of this meta-analysis revealed that CP could serve as a therapeutic option for patients exhibiting low or negative PD-L1 expression, which provided better long-term outcomes compared with chemotherapy or P. The fundamental principle of combining PD-1/PD-L1 inhibitors and CTLA-4 inhibitors is that they operate through distinct modes of action. Anti-CTLA-4 primarily targets the lymph node region, facilitating the generation and multiplication of activated T cells, while PD-1 antagonists predominantly function at the tumor periphery, inhibiting tumor progression.-infiltrating tumor PD-L1-expressing tumors and plasma-like dendritic cells from neutralizing cytotoxic T cells (40). Given that PD-1 and CTLA-4 regulate effector T-cell activation, proliferation, and function via independent yet complimentary pathways, the utilization of dual immune checkpoint inhibitors (PD-1/PD-L1 and CTLA-4) is a logical strategy to enhance antitumor immunity (7, 8). The concurrent inhibition of the PD-1/PD-L1 and CTLA-4 pathways has demonstrated additive or synergistic antitumor efficacy in prior research (42–44).

The heterogeneity was considerable in this meta-analysis, which might be caused by differences in baseline patient characteristics or study design. In order to identify potential sources of heterogeneity, subgroup analyses were performed based on PD-L1 expression, TMB, medication combinations, non-small cell lung cancer stage, median age, gender, histology and smoking status (**Table 2**, **Table 3**). The PD-L1 expression seemed to be an important sources of heterogeneity. However, since the poor sample size, the non-significant results regarding most subgroup analyses made it difficult to further analyze the source of heterogeneity.

Regarding safety, the findings revealed that the occurrence of adverse events in the CP group was lower than that in the chemotherapy group, but higher than P. The increased occurrence of AEs in the CP group, may be attributed to the expression of CTLA-4 after T-cell activation, which has the ability to down-regulate or prevent T-cell activation (45). Additionally, the cytoplasmic region of CTLA-4 contains immunoreceptor tyrosine inhibitory sequences that transmit inhibitory signals and thereby reduce the immune response (46). The most common AEs with an incidence greater than 10% included diarrhea, decreased appetite, fatigue, rash, pruritus, nausea, weight decreased, asthenia and back pain. The most prevalent grade 3–5 AEs with an incidence greater than 1% included pneumonia, febrile neutropenia, diarrhea, fatigue, rash, autoimmune hepatitis, colitis, pancreatitis, dehydration, anemia. It is advisable to formulate clinical response strategies for these high-frequency or severe AEs to offer more thorough guidance for clinical applications. Unfortunately, due to the limitations of the raw data, we failed to analyze adverse events by patient characteristics to explore if certain subgroups were more prone to specific adverse events, thereby providing personalized treatment recommendations.

The analysis of two clinical trials in the CP combination chemotherapy group was limited due to inadequate data sets. Both studies showed that while ICIs double-exempt combination chemotherapy improved OS and PFS, it also was associated with a notable rise in toxicity. Nevertheless, the limited sample size and preexisting experimental data may introduce inaccuracies, thereby necessitating further studies to substantiate the comparison of its therapeutic efficacy with CP, chemotherapy, or P. Nevertheless, it has been demonstrated that both have a coordinated impact on the enhancement of immunological sensitivity in cancer cells and the activation of effector immune cells (47). Furthermore, the inhibitory impact of chemotherapy on the impairment of the immune system establishes advantageous circumstances for the use of combination therapy with CP, so enhancing the reactive immune response against malignancies. The distinct and mutually exclusive toxicity profile of CP and chemotherapy renders them highly suitable for combination approaches (48, 49).

This meta-analysis was conducted on two experimental groups comparing CP versus chemotherapy, CP versus P, and three treatment regimens for patients with advanced non-small cell lung cancer. These regimens can be selected based on tumor characteristics to determine the most clinically individualized treatment. As a first-line treatment option for advanced non-small cell lung cancer, the combination of a PD-1/PD-L1 inhibitor plus a CTLA-4 inhibitor is both effective and well tolerated. PD-L1 expression has been summarized as negative (<1%), low-positive (1-50%), or high-positive (≥50%). Stratification of patients according to PD-L1 expression level as well as bTMB status revealed that patients who were negative for PD-L1 expression and treated with PD-1/PD-L1 inhibitors in combination with CTLA-4 inhibitors had improved OS. When PD-L1 expression was negative, PD-L1 inhibitor monotherapy improved OS but not PFS. Meanwhile, the results of the study showed that for patients with advanced non-small cell lung cancer with strongly positive PD-L1 expression, undergoing a dual-immunity combination therapy was more effective than chemotherapy in terms of both survival benefit and toxicity. In terms of AEs, PD-1/PD-L1 inhibitor monotherapy had the highest acceptable toxicity, followed by CP (dual-immunity combination therapy with ICIs), and finally chemotherapy. Taking PD-L1 expression and bTMB into account, treatment options can be divided into the following three categories: Patients with negative PD-L1 expression or low tumor mutation (bTMB <20 mut/Mb) can be considered to be preferred to ICIs combination therapy due to the susceptibility to ICI monotherapy resistance; patients with strong positive PD-L1 expression or high tumor mutation (bTMB ≥20 mut/Mb) can be considered as preferred to ICIs combination therapy in view of their susceptibility to ICI treatment and their susceptibility to ICI monotherapy. ICI therapy is sensitive, and ICI monotherapy has a low toxicity response. ICI monotherapy can be preferred, followed by ICIs combination therapy; patients with low positive PD-L1 expression, because there is no study directly showing which drug is more efficacious, can be considered first, followed by ICIs combination therapy. Because of the lack of prospective direct comparisons, the choice of treatment options should be considered on a patient-by-patient basis, with transparent communication with the patient about the advantages, costs, and risks of each option.

This meta-analysis has included the highest number of trials studying the efficacy and safety of first-line PD-1/PD-L1 inhibitor in combination with CTLA-4 inhibitor in the treatment of patients with advanced non-small cell lung cancer, to the best of our knowledge. Compared with previous meta-analyses (50), this meta-analysis included more updated trials with detailed subgroup analyses and exhaustive discussions of the results. The findings of our study provide valuable insights into the clinical outcomes of CP that contribute to both clinical practice and research in the field of advanced non-small cell lung cancer. However, there were some possible limitations in the meta-analysis. To begin with, while the meta-analysis have included the latest trials, the results may be unstable because of the limited sample size of. For example, only four trials reported outcomes of CP versus chemotherapy, and only three trials reported outcomes of CP versus P. The limited sample sizes could lead to poor statistical power and instability results. In addition, the heterogeneity was considerable in this meta-analysis, which might be caused by differences in baseline patient characteristics or study design. For example, the inclusion criteria was different among these trials. Only two trials included patients with PD-L1 expression <1%, while other trials did not. The treatment regimens in some trials were Nivolumab plus ipilimumab while Durvalumab plus tremelimuma in other trials. Though subgroup analyses were performed to identify potential sources of heterogeneity, it was still difficult to further analyze the source of heterogeneity due to the poor sample size and the non-significant results.

In conclusion, the findings of this meta-analysis revealed that CP was feasible and safe as first-line treatment for patients with advanced NSCLC. Specially, CP could serve as a therapeutic option for patients exhibiting low or negative PD-L1 expression, yielding superior long-term outcomes relative to chemotherapy or P. Additional RCTs with extended follow-up durations are required to substantiate these findings, particularly emphasizing efficacy among patients with varying PD-L1 expression levels, to enhance the stratified application of immunotherapy.

## Data Availability

The datasets presented in this study can be found in online repositories. The names of the repository/repositories and accession number(s) can be found in the article/**Supplementary Material.**
