# Supplementary material for: Efficacy and safety of first-line PD-1/PD-L1 inhibitor in combination with CTLA-4 inhibitor in the treatment of patients with advanced non-small cell lung cancer: a systemic review and meta-analysis
Source: Front Immunol. 2025 Feb 6;16:1515027. doi: 10.3389/fimmu.2025.1515027 (PMC11839650; doi:10.3389/fimmu.2025.1515027)
Supplement: Supplementary file 1 [file Table1.docx]

**Table S1** Search strategy

| PubMed | | |
| --- | --- | --- |
| No. | Query | Results |
| #1 | ((((((((((("Carcinoma, Non-Small-Cell Lung"[Mesh]) OR (Carcinoma, Non Small Cell Lung[Title/Abstract])) OR (Carcinomas, Non-Small-Cell Lung[Title/Abstract])) OR (Lung Carcinoma, Non-Small-Cell[Title/Abstract])) OR (Lung Carcinomas, Non-Small-Cell[Title/Abstract])) OR (Non-Small-Cell Lung Carcinomas[Title/Abstract])) OR (Non-Small-Cell Lung Carcinoma[Title/Abstract])) OR (Non Small Cell Lung Carcinoma[Title/Abstract])) OR (Carcinoma, Non-Small Cell Lung[Title/Abstract])) OR (Non-Small Cell Lung Carcinoma[Title/Abstract])) OR (Non-Small Cell Lung Cancer[Title/Abstract])) OR (Nonsmall Cell Lung Cancer[Title/Abstract]) | 101614 |
| #2 | (((((((((((((((((((((((((((PD-L1 inhibit[Title/Abstract]) OR (PD-L1 Inhibitors[Title/Abstract])) OR (PD L1 Inhibitors[Title/Abstract])) OR (PD-L1 Inhibitor[Title/Abstract])) OR (PD L1 Inhibitor[Title/Abstract])) OR (Programmed Death-Ligand 1 Inhibitors[Title/Abstract])) OR (Programmed Death Ligand 1 Inhibitors[Title/Abstract])) OR (Atezolizumab[Title/Abstract])) OR (Durvalumab[Title/Abstract])) OR (Avelumab[Title/Abstract])) OR (PD-1 Inhibitors[Title/Abstract])) OR (PD 1 Inhibitors[Title/Abstract])) OR (PD-1 Inhibitor[Title/Abstract])) OR (Inhibitor, PD-1[Title/Abstract])) OR (PD 1 Inhibitor[Title/Abstract])) OR (Programmed Cell Death Protein 1 Inhibitor[Title/Abstract])) OR (Programmed Cell Death Protein 1 Inhibitor[Title/Abstract])) OR (Nivolumab[Title/Abstract])) OR (Pembrolizumab[Title/Abstract])) OR (Toripalimab[Title/Abstract])) OR (Tislelizumab[Title/Abstract])) OR (Camrelizumab[Title/Abstract])) OR (GLS-010[Title/Abstract])) OR (Cemiplimab[Title/Abstract])) OR (Sintilimab[Title/Abstract])) OR (Zimberelimab[Title/Abstract])) OR (Prolgolimab[Title/Abstract])) OR (Dostarlimab[Title/Abstract]) | 23807 |
| #3 | ((((((((((((((((CTLA-4 Inhibitors[Title/Abstract]) OR (CTLA 4 Inhibitors[Title/Abstract])) OR (CTLA-4 Inhibitor[Title/Abstract])) OR (CTLA 4 Inhibitor[Title/Abstract])) OR (Cytotoxic T-Lymphocyte-Associated Protein 4 Inhibitors[Title/Abstract])) OR (Cytotoxic T Lymphocyte Associated Protein 4 Inhibitors[Title/Abstract])) OR (Cytotoxic T-Lymphocyte-Associated Protein 4 Inhibitor[Title/Abstract])) OR (Cytotoxic T Lymphocyte Associated Protein 4 Inhibitor[Title/Abstract])) OR (Ipilimumab[Title/Abstract])) OR (Tremelimumab[Title/Abstract])) OR (IB-310[Title/Abstract])) OR (XTX-101[Title/Abstract])) OR (BMS-986249[Title/Abstract])) OR (Quavonlimab[Title/Abstract])) OR (AGEN-1181[Title/Abstract])) OR (YH-001[Title/Abstract])) OR (Zalifrelimab[Title/Abstract]) | 5781 |
| #4 | #2 AND #3 | 3712 |
| #5 | (randomized controlled trial [pt] OR controlled clinical trial [pt] OR randomized [tiab] OR placebo [tiab] OR clinical trials as topic [mesh:noexp] OR randomly [tiab] OR trial [ti]) NOT (animals [mh] NOT humans [mh]) | 1462006 |
| #6 | #1 AND #4 AND #5 | 123 |

| Embase | | |
| --- | --- | --- |
| No. | Query | Results |
| #1 | 'non small cell lung cancer'/exp OR 'non small cell lung cancer' OR 'bronchial non small cell cancer':ti,ab,kw OR 'bronchial non small cell carcinoma':ti,ab,kw OR 'carcinoma, non-small-cell lung':ti,ab,kw OR 'lung cancer, non small cell':ti,ab,kw OR 'lung non small cell cancer':ti,ab,kw OR 'lung non small cell carcinoma':ti,ab,kw OR 'non oat cell lung cancer':ti,ab,kw OR 'non small cell bronchial cancer':ti,ab,kw OR 'non small cell cancer, lung':ti,ab,kw OR 'non small cell lung carcinoma':ti,ab,kw OR 'non small cell pulmonary cancer':ti,ab,kw OR 'non small cell pulmonary carcinoma':ti,ab,kw OR 'non squamous nsclc':ti,ab,kw OR 'non-oat cell lung cancer':ti,ab,kw OR 'non-small-cell lung carcinoma':ti,ab,kw OR 'nonsmall cell carcinoma of the lung':ti,ab,kw OR 'nonsmall cell lung cancer':ti,ab,kw OR 'nonsmall cell lung carcinoma':ti,ab,kw OR 'pulmonary non small cell cancer':ti,ab,kw OR 'pulmonary non small cell carcinoma':ti,ab,kw OR 'non small cell lung cancer':ti,ab,kw | 233126 |
| #2 | ‘pd l1 inhibitor’/exp OR ‘Durvalumab’:ti,ab,kw OR ‘Nivolumab’:ti,ab,kw OR ‘Atezolizumab’:ti,ab,kw OR ‘Avelumab’:ti,ab,kw OR ‘pd 1 inhibitor’/exp OR ‘Pembrolizumab’:ti,ab,kw OR ‘Toripalimab’:ti,ab,kw OR ‘Tislelizumab’:ti,ab,kw OR ‘Camrelizumab’:ti,ab,kw OR ‘GLS-010’:ti,ab,kw OR ‘Cemiplimab’:ti,ab,kw OR ‘Sintilimab’:ti,ab,kw OR ‘Zimberelimab’:ti,ab,kw OR ‘Prolgolimab’:ti,ab,kw OR ‘Dostarlimab’:ti,ab,kw | 45780 |
| #3 | ‘cytotoxic T lymphocyte antigen 4 antibody’/exp 'CD152 antibody':ti,ab,kw OR 'CTLA 4 antibody':ti,ab,kw OR 'CTLA4 antibody':ti,ab,kw OR 'cytotoxic T lymphocyte antigen 4 antibody':ti,ab,kw OR ‘Ipilimumab’:ti,ab,kw OR ‘Tremelimumab’:ti,ab,kw OR ‘IB-310’:ti,ab,kw OR ‘XTX-101’:ti,ab,kw OR ‘BMS-986249’:ti,ab,kw OR ‘Quavonlimab’:ti,ab,kw OR ‘AGEN-1181’:ti,ab,kw OR ‘YH-001’:ti,ab,kw OR ‘Zalifrelimab’:ti,ab,kw | 13059 |
| #4 | #2 AND #3 | 8427 |
| #5 | 'randomized controlled trial'/exp OR 'controlled trial, randomized':ti,ab,kw OR 'randomised controlled study':ti,ab,kw OR 'randomised controlled trial':ti,ab,kw OR 'randomized controlled study':ti,ab,kw OR 'trial, randomized controlled':ti,ab,kw | 820113 |
| #6 | #1 AND #4 AND #5 | 256 |

| cochrane library | | |
| --- | --- | --- |
| No. | Query | Results |
| #1 | (Non-Small-Cell Lung Carcinomas OR Lung Carcinomas, Non-Small-Cell OR Nonsmall Cell Lung Cancer OR Non-Small Cell Lung Carcinoma OR Carcinomas, Non-Small-Cell Lung OR Carcinoma, Non Small Cell Lung OR Lung Carcinoma, Non-Small-Cell OR Non Small Cell Lung Carcinoma OR Non-Small-Cell Lung Carcinoma OR Carcinoma, Non-Small Cell Lung OR Non-Small Cell Lung Cancer):ab,ti,kw | 16380 |
| #2 | (Cytotoxic T-Lymphocyte-Associated Protein 4 Inhibitor OR Cytotoxic T Lymphocyte Associated Protein 4 Inhibitors OR CTLA 4 Inhibitors OR CTLA 4 Inhibitor OR Cytotoxic T Lymphocyte Associated Protein 4 Inhibitor OR CTLA-4 Inhibitor OR CTLA-4 Inhibitors OR Cytotoxic T-Lymphocyte-Associated Protein 4 Inhibitors OR Ipilimumab OR Tremelimumab OR IB-310 OR XTX-101 OR BMS-986249 OR Quavonlimab OR AGEN-1181 OR YH-001 OR Zalifrelimab):ti,ab,kw | 2377 |
| #3 | (Programmed Death Ligand 1 Inhibitors OR Programmed Death-Ligand 1 Inhibitors OR PD-L1 Inhibitors OR PD L1 Inhibitors OR PD L1 Inhibitor OR PD-L1 Inhibitor OR Atezolizumab OR Durvalumab OR Avelumab OR PD-1 Inhibitors OR PD-1 Inhibitor OR PD 1 Inhibitor OR Programmed Cell Death Protein 1 Inhibitor OR Inhibitor, PD-1 OR Programmed Cell Death Protein 1 Inhibitors OR PD 1 Inhibitors OR Pembrolizumab OR Nivolumab OR Toripalimab OR Tislelizumab OR Camrelizumab OR GLS-010 OR Cemiplimab OR Sintilimab OR Zimberelimab OR Prolgolimab OR Dostarlimab):ab,ti,kw | 13899 |
| #4 | #2 AND #3 | 1942 |
| #5 | (Randomized Controlled Trial OR Controlled Clinical Trial OR Randomized OR Placebo OR Clinical Trials As Topic OR Randomly OR Trial):ab,ti,kw | 1480900 |
| #6 | #1 AND #4 AND #5 | 356 |

| Web of science | | |
| --- | --- | --- |
| No. | Query | Results |
| #1 | ((((((((((TS=(Carcinoma, Non Small Cell Lung)) OR TS=(Carcinomas, Non-Small-Cell Lung)) OR TS=(Lung Carcinoma, Non-Small-Cell)) OR TS=(Lung Carcinomas, Non-Small-Cell)) OR TS=(Non-Small-Cell Lung Carcinomas)) OR TS=(Non-Small-Cell Lung Carcinoma)) OR TS=(Non Small Cell Lung Carcinoma)) OR TS=(Carcinoma, Non-Small Cell Lung)) OR TS=(Non-Small Cell Lung Carcinoma)) OR TS=(Non-Small Cell Lung Cancer)) OR TS=(Nonsmall Cell Lung Cancer) | 116530 |
| #2 | (((((((((((((((((((((((((((TS=(PD-L1 inhibit)) OR TS=(PD-L1 Inhibitors)) OR TS=(PD L1 Inhibitors)) OR TS=(PD-L1 Inhibitor)) OR TS=(PD L1 Inhibitor)) OR TS=(Programmed Death-Ligand 1 Inhibitors)) OR TS=(Programmed Death Ligand 1 Inhibitors)) OR TS=(Durvalumab)) OR TS=(Atezolizumab)) OR TS=(Avelumab)) OR TS=(PD-1 Inhibitors)) OR TS=(PD 1 Inhibitors)) OR TS=(PD-1 Inhibitor)) OR TS=(Inhibitor, PD-1)) OR TS=(PD 1 Inhibitor)) OR TS=(Programmed Cell Death Protein 1 Inhibitor)) OR TS=(Programmed Cell Death Protein 1 Inhibitors)) OR TS=(Nivolumab)) OR TS=(Pembrolizumab)) OR TS=(Toripalimab)) OR TS=(Tislelizumab)) OR TS=(Camrelizumab)) OR TS=(GLS-010)) OR TS=(Cemiplimab)) OR TS=(Sintilimab)) OR TS=(Zimberelimab)) OR TS=(Prolgolimab)) OR TS=(Dostarlimab) | 66967 |
| #3 | ((((((((((((((((TS=(CTLA-4 Inhibitors)) OR TS=(CTLA 4 Inhibitors)) OR TS=(CTLA-4 Inhibitor)) OR TS=(CTLA 4 Inhibitor)) OR TS=(Cytotoxic T-Lymphocyte-Associated Protein 4 Inhibitors)) OR TS=(Cytotoxic T Lymphocyte Associated Protein 4 Inhibitors)) OR TS=(Cytotoxic T-Lymphocyte-Associated Protein 4 Inhibitor)) OR TS=(Cytotoxic T Lymphocyte Associated Protein 4 Inhibitor)) OR TS=(Ipilimumab)) OR TS=(Tremelimumab)) OR TS=(IB-310)) OR TS=(XTX-101)) OR TS=(BMS-986249)) OR TS=(Quavonlimab)) OR TS=(AGEN-1181)) OR TS=(YH-001)) OR TS=(Zalifrelimab) | 16530 |
| #4 | #2 AND #3 | 11767 |
| #5 | ((((((TS=(randomized controlled trial)) OR TS=(controlled clinical tria)) OR TS=(randomized)) OR TS=(placebo)) OR TS=(clinical trials as topic)) OR TS=(randomly)) OR TS=(trial) | 2698916 |
| #6 | #1 AND #4 AND #5 | 655 |

**Table S2** All-grade adverse events in included studies

| **Adverse events** | **CP group** | | |  | **Chemotherapy group** | | |  | **P group** | | |
| --- | --- | --- | --- | --- | --- | --- | --- | --- | --- | --- | --- |
|  | **Studies involved** | **Event/total** | **%** |  | **Studies involved** | **Event/total** | **%** |  | **Studies involved** | **Event/total** | **%** |
| **Blood and lymphatic system disorders** | | | | | | | | | | | |
| Anemia | 4 | 92/1197 | 7.68 |  | 4 | 412/1110 | 37.12 |  | 3 | 52/767 | 6.78 |
| Bone marrow failure | - | - | - |  | 1 | 6/78 | 7.69 |  | - | - | - |
| Febrile neutropenia | - | - | - |  | - | - | - |  | - | - | - |
| Neutropenia | 4 | 39/1197 | 3.26 |  | 4 | 250/1110 | 22.52 |  | 1 | 6/369 | 16.26 |
| Thrombocytopenia | 2 | 23/544 | 4.23 |  | 3 | 93/540 | 17.22 |  | 2 | 7/486 | 1.44 |
| **Gastrointestinal disorders** | | | | | | | | | | | |
| Colitis | - | - | - | - | - | - | - |  | - | - | - |
| Constipation | 4 | 111/1197 | 9.27 |  | 4 | 189/1110 | 17.03 |  | 2 | 56/650 | 8.62 |
| Diarrhea | 4 | 208/1197 | 17.38 |  | 4 | 119/1110 | 10.72 |  | 3 | 86/767 | 11.21 |
| Nausea | 4 | 154/1197 | 12.87 |  | 4 | 402/1110 | 32.22 |  | 3 | 60/767 | 7.82 |
| Vomiting | 4 | 69/1197 | 5.76 |  | 4 | 166/1110 | 14.95 |  | 3 | 37/767 | 4.82 |
| **General disorders** | | | | | | | | | | | |
| Asthenia | 4 | 129/1197 | 10.78 |  | 4 | 123/1110 | 11.08 |  | 3 | 72/767 | 9.39 |
| Back pain | 1 | 38/371 | 10.24 |  | 1 | 32/352 | 9.09 |  | 2 | 69/650 | 10.62 |
| Fatigue | 4 | 184/1197 | 15.37 |  | 4 | 214/1110 | 19.28 |  | 3 | 84/767 | 10.95 |
| Pyrexia | 3 | 8/621 | 1.29 |  | 3 | 2/540 | 0.37 |  | 3 | 69/767 | 9.00 |
| Weight decreased | 2 | 49/448 | 10.94 |  | 2 | 21/430 | 4.88 |  | 1 | 39/369 | 10.57 |
| **Investigations** | | | | | | | | | | | |
| Alanine aminotransaminase increased | 3 | 48/608 | 7.89 |  | 3 | 40/537 | 7.49 |  | 1 | 3/117 | 2.56 |
| Aspartate aminotransferase  increased | 2 | 24/250 | 9.60 |  | 2 | 25/188 | 13.30 |  | 1 | 2/117 | 1.71 |
| Amylase increased | 2 | 40/435 | 9.20 |  | 2 | 8/427 | 1.87 |  | - | - | - |
| Decreased platelet count | - | - | - | - | - | - | - |  | - | - | - |
| Lipase increased | 2 | 39/435 | 8.97 |  | 2 | 5/427 | 1.17 |  | - | - | - |
| Neutrophil count decreased | 2 | 18/531 | 3.39 |  | 2 | 31/459 | 6.75 |  | - | - | - |
| White blood cell count | 3 | 14/608 | 2.30 |  | 3 | 54/537 | 10.06 |  | 1 | 1/117 | 0.85 |
| **Metabolism and nutrition disorders** | | | | | | | | | | | |
| Decreased appetite | 4 | 201/1197 | 16.79 |  | 4 | 234/1110 | 21.08 |  | 3 | 70/767 | 9.13 |
| Dehydration | - | - | - | - | - | - | - |  | - | - | - |
| Hepatotoxicity | 1 | 10/358 | 2.79 |  | 1 | 2/349 | 0.57 |  | - | - | - |
| **Skin and subcutous tissue disorders** | | | | | | | | | | | |
| Rash | 4 | 181/1197 | 15.12 |  | 4 | 90/1110 | 8.11 |  | 3 | 74/767 | 9.65 |
| Pruritus | 4 | 175/1197 | 14.12 |  | 4 | 30/1110 | 2.70 |  | 3 | 95/767 | 12.39 |

**Abbreviation**: AEs: adverse events; CP: CTLA4 inhibitor plus PD1/PDL1 inhibitor; GGT: Gamma-glutamyltransferase; P: PD1/PDL1 inhibitor

**Table S3** Grade 3-5 adverse events in included studies

| **Adverse events** | **CP group** | | |  | **Chemotherapy group** | | |  | **P group** | | |
| --- | --- | --- | --- | --- | --- | --- | --- | --- | --- | --- | --- |
|  | **Studies involved** | **Event/total** | **%** |  | **Studies involved** | **Event/total** | **%** |  | **Studies involved** | **Event/total** | **%** |
| **Blood and lymphatic system disorders** | | | | | | | | | | | |
| Anemia | 4 | 15/1197 | 1.25 |  | 4 | 124/1110 | 11.17 |  | 3 | 7/767 | 0.91 |
| Bone marrow failure | - | - | - |  | 1 | 3/78 | 3.85 |  | - | - | - |
| Febrile neutropenia | 1 | 11/358 | 3.07 |  | 2 | 11/701 | 1.57 |  | - | - | - |
| Neutropenia | 4 | 1/1197 | 0.09 |  | 4 | 107/1110 | 9.64 |  | - | - | - |
| Pancytopenia | - | - | - |  | 1 | 6/352 | 1.70 |  | 1 | 1/117 | 0.85 |
| Thrombocytopenia | 3 | 7/621 | 1.23 |  | 3 | 22/603 | 3.69 |  | - | - | - |
| **Cardiac disorders** | | | | | | | | | | | |
| Acute myocardial infarctio | 1 | 2/468 | 0.43 |  | 1 | 1/110 | 0.90 |  | - | - | - |
| Atrial fibrillation | 2 | 7/729 | 0.96 |  | 3 | 4/819 | 4.94 |  | 1 | 2/396 | 0.51 |
| Cardiac arrest | 2 | 2/729 | 0.27 |  | 1 | 2/349 | 0.57 |  | 2 | 2/650 | 0.31 |
| Cardiac failure | 2 | 7/544 | 1.29 |  | - | - | - |  | 1 | 3/117 | 2.56 |
| Coronary artery disease | - | - | - |  | 4 | 1/78 | 1.28 |  | - | - | - |
| Myocardial infarction | 1 | 2/371 | 0.54 |  | - | - | - |  | - | - | - |
| Pericarditis | 2 | 2/544 | 0.37 |  | - | - | - |  | 1 | 1/369 | 0.27 |
| **Endocrine disorders** | | | | | | | | | | | |
| Adrenal insufficiency | 2 | 3/544 | 0.55 |  | - | - | - |  | 2 | 4/650 | 0.62 |
| Hypophysitis | 1 | 2/371 | 0.54 |  | - | - | - |  | 1 | 1/281 | 0.36 |
| Endocrine disorder | - | - | - |  | - | - | - |  | - | - | - |
| **Gastrointestinal disorders** | | | | | | | | | | | |
| Colitis | 2 | 9/544 | 1.65 |  | - | - | - |  | 2 | 3/650 | 0.46 |
| Constipation | 1 | 2/371 | 0.54 |  | 1 | 1/349 | 0.28 |  | 2 | 3/650 | 0.46 |
| Diarrhea | 4 | 26/1197 | 2.17 |  | 4 | 12/1110 | 1.08 |  | 3 | 3/767 | 0.39 |
| Duodenitis | - | - | - |  | - | - | - |  | - | - | - |
| Dysphagia | 2 | 2/250 | 0..01 |  | 1 | 1/110 | 0.90 |  | 3 | 4/767 | 0.52 |
| Enterocolitis | 2 | 3/544 | 0.55 |  | 2 | 2/451 | 0.44 |  | - | - | - |
| Faecaloma | - | - | - |  | 1 | 1/352 | 0.28 |  | - | - | - |
| Food poisoning | - | - | - |  | - | - | - |  | - | - | - |
| Nausea | 3 | 8/1024 | 0.78 |  | 3 | 20/1000 | 0.02 |  | - | - | - |
| Pancreatitis | 1 | 2/173 | 1.56 |  | - | - | - |  | - | - | - |
| Vomiting | 4 | 4/1197 | 0.33 |  | 4 | 23/1110 | 2.07 |  | 1 | 1/281 | 0.36 |
| **General disorders** | | | | | | | | | | | |
| Asthenia | 2 | 3/544 | 0.55 |  | - | - | - |  | 1 | 33/281 | 11.74 |
| Death | 2 | 2/544 | 0.37 |  | 1 | 1/352 | 0.28 |  | 2 | 5/650 | 0.77 |
| Fatigue | 3 | 24/1120 | 2.14 |  | 3 | 20/1032 | 1.94 |  | 3 | 12/767 | 1.56 |
| General physical health deterioration | 1 | 3/371 | 0.81 |  | 1 | 2/352 | 0.59 |  | 2 | 2/650 | 0.31 |
| Pain | 1 | 1/173 | 0.58 |  | - | - | - |  | - | - | - |
| Perforation | - | - | - |  | - | - | - |  | - | - | - |
| Pyrexia | 2 | 1/205 | 0.49 |  | 2 | 2/118 | 1.69 |  | 3 | 8/767 | 1.04 |
| Sudden cardiac death | 2 | 4/544 | 0.74 |  | 1 | 4/110 | 3.64 |  | 2 | 2/486 | 0.41 |
| **Hepatobiliary disorders** | | | | | | | | | | | |
| Autoimmune hepatitis | 1 | 3/173 | 1.73 |  | - | - | - |  | - | - | - |
| Drug-induced liver injury | 2 | 6/421 | 1.42 |  | - | - | - |  | 2 | 2/486 | 0.41 |
| Hepatitis | 3 | 3/544 | 0.55 |  | - | - | - |  | 2 | 2/650 | 0.31 |
| **Immune system disorders** | | | | | | | | | | | |
| Contrast media reaction | - | - | - |  | - | - | - |  | - | - | - |
| Drug hypersensitivity | - | - | - |  | - | - | - |  | 1 | 1/369 | 0.27 |
| **Infections and infestations** | | | | | | | | | | | |
| Appendicitis | 1 | 1/173 | 0.58 |  | 1 | 1/110 | 0.91 |  | 1 | 29/281 | 10.32 |
| Cellulitis | - | - | - |  | 1 | 3/352 | 0.85 |  | 2 | 3/650 | 0.46 |
| Empyema | - | - | - | - | 1 | 1/352 | 0.28 |  | 1 | 1/281 | 0.36 |
| Escherichia infection | 1 | 1/173 | 0.58 |  | - | - | - |  | - | - | - |
| Gastroenteritis | 3 | 3/621 | 0.48 |  | - | - | - |  | 1 | 3/281 | 1.07 |
| Herpes zoster | 1 | 1/371 | 0.27 |  | - | - | - |  | 1 | 1/369 | 0.27 |
| Infection | 1 | 1/173 | 0.58 |  | - | - | - |  | 1 | 1/369 | 0.27 |
| Lower respiratory tract infection | - | - | - |  | 1 | 1/110 | 0.91 |  | 1 | 1/281 | 0.36 |
| Lung abscess | - | - | - |  | 1 | 1/349 | 0.28 |  | - | - | - |
| Lung infection | 1 | 1/371 | 0.28 |  | - | - | - |  | 1 | 1/369 | 0.27 |
| Pneumonia | 3 | 32/1024 | 3.13 |  | 3 | 11/1000 | 0.01 |  | 3 | 47/767 | 6.13 |
| Pneumonia bacterial | - | - | - |  | 1 | 1/110 | 0.91 |  | - | - | - |
| Respiratory tract infection | 3 | 2/621 | 0.32 |  | 3 | 3/540 | 0.56 |  | 1 | 1/117 | 0.85 |
| Sepsis | 1 | 3/371 | 0.81 |  | 1 | 2/352 | 0.57 |  | 3 | 5/767 | 0.65 |
| Septic shock | 1 | 2/371 | 0.54 |  | 1 | 2/352 | 0.57 |  | 2 | 4/650 | 0.66 |
| Urinary tract infection | 1 | 1/371 | 0.27 |  | 1 | 3/352 | 0.85 |  | - | - | - |
| **Injury, poisoning and procedural complications** | | | | | | | | | | | |
| Femur fracture | - | - | - |  | 1 | 1/352 | 0.28 |  | 2 | 3/399 | 0.75 |
| Head injury | 3 | 3/621 | 0.48 |  | - | - | - |  | - | - | - |
| **Investigations** | | | | | | | | | | | |
| Blood creatinine increased | 2 | 2/544 | 0.37 |  | 1 | 1/78 | 1.28 |  | 1 | 1/369 | 0,27 |
| C-reactive protein increased | 1 | 1/173 | 0.58 |  | 1 | 2/352 | 0.57 |  | - | - | - |
| Haematocrit decreased | 1 | 1/371 | 0.27 |  | 1 | 1/352 | 0.28 |  | - | - | - |
| Neutrophil count decreased | - | - | - |  | 2 | 4/430 | 0.93 |  | - | - | - |
| Leukopenia | 3 | 4/621 | 0.64 |  | 3 | 61/603 | 10.12 |  | 2 | 2/486 | 0.41 |
| **Metabolism and nutrition disorders** | | | | | | | | | | | |
| Decreased appetite | 4 | 7/1197 | 0.58 |  | 4 | 11/1110 | 0.99 |  | 1 | 1/369 | 0.27 |
| Dehydration | 1 | 5/371 | 1.35 |  | 1 | 1/352 | 0.28 |  | 1 | 1/117 | 0.85 |
| Hypokalaemia | 2 | 2/250 | 0.01 |  | 1 | 1/352 | 0.28 |  | - | - | - |
| Hyponatraemia | - | - | - |  | 1 | 1/352 | 0.28 |  | 1 | 1/369 | 0.27 |
| Type 2 diabetes mellitus | 3 | 3/806 | 0.37 |  | - | - | - |  | - | - | - |
| **Skin and subcutaneous tissue disorders** | | | | | | | | | | | |
| Rash | 3 | 14/806 | 1.74 |  | - | - | - |  | - | - | - |
| Toxic skin eruption | 1 | 1/371 | 0.27 |  | - | - | - |  | - | - | - |
| **Vascular disorders** | | | | | | | | | | | |
| Deep vein thrombosis | 2 | 2/729 | 0.27 |  | - | - | - |  | 1 | 1/369 | 0.27 |
| Embolism | 2 | 2/544 | 0.37 |  | - | - | - |  | 1 | 4/177 | 2.26 |
| Hypotension | - | - | - |  | 2 | 2/549 | 0.36 |  | 1 | 1/281 | 0.36 |
| Superior vena cava syndrome | 2 | 2/621 | 0.32 |  | - | - | - |  | 2 | 3/650 | 0.46 |

**Abbreviation**: AEs: adverse events; CP: CTLA4 inhibitor plus PD1/PDL1 inhibitor; GGT: Gamma-glutamyltransferase; P: PD1/PDL1 inhibitor
